# Effects of online stigma‐reduction programme for people experiencing mental health conditions: A systematic review and meta‐analysis

**DOI:** 10.1111/inm.12893

**Published:** 2021-06-03

**Authors:** Yong‐Shian Goh, Qing Yun Jenna Ow Yong, Wai‐San Wilson Tam

**Affiliations:** ^1^ Alice Lee Centre for Nursing Studies Yong Loo Lin School of Medicine National University of Singapore Singapore; ^2^ Alice Lee Centre for Nursing Studies National University Health System Singapore

**Keywords:** face‐to‐face stigma reduction program, meta‐analysis, online stigma reduction program, People experiencing mental health conditons, systematic review

## Abstract

Despite the increased awareness of mental health‐related issues, people experiencing mental health conditions have continued to face stigmatization worldwide. The literature on help‐seeking behaviours has frequently highlighted the development of self‐stigma because of public stigma and emphasized the need to address public stigmatization faced by them. Given the increasing acceptance of digital services in recent years, this systematic review aimed to examine the effects of online and face‐to‐face anti‐stigma interventions in reducing public stigma towards people experiencing mental health conditions. A search was conducted on the Cochrane Library, CINAHL, PubMed, Embase, PsycInfo, and ProQuest from inception of the databases to October 2020. Studies were included in this review if they have explored: (i) public stigmatization towards people of all ages with different types of mental health conditions; (ii) online interventions; and (iii) face‐to‐face interventions. Nine studies were included in this review, of which only five were included in the meta‐analysis as the remaining four had incomplete data. The meta‐analysis included an aggregate of 1203 participants while the four excluded studies included 713 participants. Results revealed that online interventions performed favourably with small effect sizes in comparison to face‐to‐face, wait‐list control, and no‐intervention groups. Results from the studies excluded from the meta‐analysis also found a significant reduction of public stigmatization with online interventions. Such findings provide insightful evidence for the effectiveness of online interventions in reducing public stigmatization. Hence, mental health organizations and groups can consider adopting online interventions suitable for their target audience and type of mental health conditions.

## Introduction

The global prevalence of mental health conditions has remained consistently high across the past few decades (Richter *et al*. 2019), with an estimated 29.2% of the population experiencing at least one form of mental health condition in their lifetime (Steel *et al*. 2014). In the United States of America (USA) alone, 51.5 million adults (20.6%) had at least one form of mental health condition in 2019 (Substance Abuse & Mental Health Services Administration [SAMHSA] 2020). Yet, only 44.8% (23 million adults) of people experiencing mental health conditions (PMHCs) have received mental health services (SAMHSA 2020). This thus suggests the presence of a significant gap in accessing treatment for mental health conditions. While structural barriers including the socio‐economic status, governmental policies, and availability and accessibility of mental health services may underlie the gap, prevalent attitude‐related barriers such as stigmatization also discourage the use of existing mental health services, thus exacerbating the gap (Herrera‐Ferrá 2020).

Stigma is conceptualized as an attribute that discredits an individual, typically invoking a sense of shame, self‐blame, and social exclusion (Goffman 1963; Waqas *et al*. 2020). Despite the focus on reducing stigma related to mental health conditions over the last few decades, PMHCs have continued to experience much stigmatization worldwide, as reported in China (Young & Ng 2016), Greece (Tzouvara *et al*. 2016), Canada (Weeks *et al*. 2017), Nepal (Maharjan & Panthee 2019), and the USA (Bonfils *et al*. 2018). Dire consequences commonly faced by PMHCs include social isolation (Linz & Sturm 2013), unemployment (Brouwers 2020), reduced access to health care (Fraser *et al*. 2020), impaired quality of life (Świtaj *et al*. 2017), and feelings of hopelessness, shame, and guilt (Oexle *et al*. 2017). These may result in delayed treatment‐seeking behaviours (Kular *et al*. 2018) or even suicidal ideations (Oexle *et al*. 2017) among the PMHCs.

Stigma related to mental health conditions typically presents in two main forms: public stigma and self‐stigma. Public stigma refers to societal stigmatizing reactions and attitudes where PMHCs are perceived to be dangerous or incompetent, or to have a character weakness (Rogers 2018) whereas self‐stigma refers to an internalized feeling of shame, guilt, and low self‐regard due to societal prejudice (Waqas *et al*. 2020). Help‐seeking literature has thus far underlined self‐stigma as a development of anticipated or experienced public stigma to varying degrees, depending on the mental health condition (Hing & Russell 2017; Vogel *et al*. 2013). This is supported by the modified labelling theory (Link *et al*. 1989) which postulates that external perceptions of PMHCs and negative labels attached to mental illnesses are internalized by PMHCs which later influence their self‐image and lead to self‐stigma. Hence, addressing the public stigmatization of PMHCs is paramount in bridging the mental health treatment gap.

Traditional interventions targeting public stigma are often conducted in person at educational institutions, military bases, workplaces, and healthcare organizations, and employ strategies such as psychoeducation workshops, social activism in the form of protests, contact‐based approaches, and reading programmes (Corrigan 2012; Moll *et al*. 2015; Yamaguchi *et al*. 2013a). Reviews on anti‐stigma interventions (Corrigan *et al*. 2012; Griffiths *et al*. 2014; Yamaguchi *et al*. 2013b) have found that educational and contact‐based approaches were effective in improving knowledge of mental health conditions, reducing stigma, improving attitudes, and reducing feelings of social distancing towards PMHCs. Despite their findings, existing reviews have not examined how the modality of the interventions would affect their effectiveness. Furthermore, given the increasing prevalence of information technology – reflected by an estimated 87% Internet usage rate (International Telecommunication Union 2020) and 98% 3G mobile network coverage in developed countries in 2019 – an online platform will be ideal for a widespread, low‐cost outreach to the population (Rogers 2018). This surge in the interest in and acceptance of digital services has been further catalysed by the COVID‐19 pandemic (Wind *et al*. 2020), due to which safe‐distancing measures have been integrated as part of our daily lives. Therefore, an empirical assessment of the effects of online interventions for reducing stigmatization of mental health conditions could offer insights upon which mental health nurses can act in years to come.

## Aims

This review aimed to examine the effects of online anti‐stigma interventions when compared with non‐online interventions in reducing public stigma towards people experiencing mental health conditions. The specific review question identified were as follows: (1) How effective are online anti‐stigma interventions in reducing public stigma when compared with face‐to‐face anti‐stigma interventions?

## Method

### Search strategy

A review protocol was developed and registered on PROSPERO (CRD42020213165) according to recommendations of the Preferred Reporting Items for Systematic Reviews and Meta‐analysis (PRISMA) statement (Moher *et al*. 2015) to ensure methodological rigour of the systematic review (Tam *et al*. 2019). A search strategy was formulated to identify studies on anti‐stigma interventions targeting public stigma towards PMHCs. To maximize the retrieval of relevant studies, help was enlisted from an experienced librarian. All available and relevant primary studies were then identified through the search with specific keywords and Medical Subject Heading (MeSH) terms from inception of the databases to 16 October 2020 in order to ensure the inclusion of updated materials in clinical practices (Tam *et al*. 2017). The keywords included ‘Social Stigma’ [MeSH], ‘Social Distance’ [MeSH], ‘Social Rejection’ [MeSH], ‘Mental Disorders’ [MeSH], and ‘Internet‐based intervention’ [MeSH]. Through Boolean search terms, five databases (CINAHL, PubMed, Embase, PsycInfo, and the Cochrane Library) and ProQuest were included, given their coverage of different disciplines including medicine (Pubmed, Embase, and the Cochrane Library), psychology (PsycInfo), nursing, and allied health (CINAHL). End‐reference lists of the identified studies were hand‐searched to ensure no omission of additional studies (Whittemore & Knafl 2005). The review was limited to articles in English, as the team had no access to interpreters.

### Eligibility criteria

#### Population

The eligible studies had participants of all ages from the general population.

#### Intervention

The eligible studies examined online interventions targeted at reducing public stigma towards people experiencing mental health conditions.

#### Comparator

Studies with no comparator, a passive comparator (usual care or wait‐list control group) or active comparator (other face‐to‐face interventions) were included.

##### Outcomes

The primary outcome for this review was stigmatizing attitudes towards people experiencing mental health conditions. The secondary outcomes were threefold: stigma experienced by people with different mental health conditions; knowledge of mental health conditions; and the desire for social contact with PMHCs. Studies were excluded if they had measured only internalized or self‐stigma.

### Selection of articles

All retrieved records from database search were uploaded into EndNote X9, and duplicate studies were removed electronically. For the remaining studies, the titles and abstracts were reviewed independently by YSG and JOY; studies not meeting the inclusion and exclusion criteria were removed at this stage. Potential articles deemed suitable by at least one author were then downloaded for further scrutiny. The articles were then reviewed independently by YSG and JOY and disagreements were consensually resolved through discussion with the third reviewer (TWSW).

### Data extraction

Data extraction was then performed independently by YSG and JOY for the following aspects: study‐related information (author, location, year, research design, and sample size); and primary outcome (stigma attitudes). The authors of the articles were also contacted for missing data on numerical outcomes. To ensure consensus when extracting information from the retrieved studies, a pilot review was conducted independently by YSG and JOY based on a data extraction form adapted from the Cochrane Handbook for Systematic Reviews of Interventions (Higgins 2020). All disagreement during data extraction was resolved in consultation with a third reviewer (WWT).

### Risk of bias

The risk of bias (ROB) of the included studies was evaluated independently by YSG and JOY based on the Cochrane Risk of Bias assessment tool (Higgins 2020). The individual domains of bias in the ROB assessment tool were rated as ‘low risk’, ‘high risk’, or ‘unclear risk’. Accordingly, a given study was rated as ‘high risk’ if more than half of its domains were evaluated to be at ‘high risk’ or ‘unclear risk’. Disagreement was consensually resolved through discussion with the third reviewer (TWSW). Review Manager 5.4 software was used to generate the ROB summary graph to illustrate the risk for the domains of each study (RevMan 2020).

### Data analysis

Post‐programme measurements for each of the outcome measures were extracted from the randomized controlled trials (RCTs) for comparison. The mean difference (MD) and its 95% confidence intervals (CIs) were computed as the effect measure. Heterogeneity was examined by the Chi‐square test and *I* ^2^ statistics. The statistical significance of heterogeneity was set at *P* < 0.10, and the degree of variability was estimated through *I* ^2^ values, with 75%, 50%, 25%, and 10%, respectively, indicating high, moderate, low, and no heterogeneity (Higgins 2020). The fixed‐effects model was used for homogeneous studies (chi‐square *P* > 0.10 & *I* ^2^ value < 50%); otherwise, the DerSimonian–Laird random‐effects model was used. A funnel plot was created to explore potential publication bias when there were 10 or more studies for the outcome (Sterne *et al*. 2011). Data were analysed through Review Manager 5.4 (RevMan 2020) according to the modality of the interventions.

## Results

**Figure 1:**
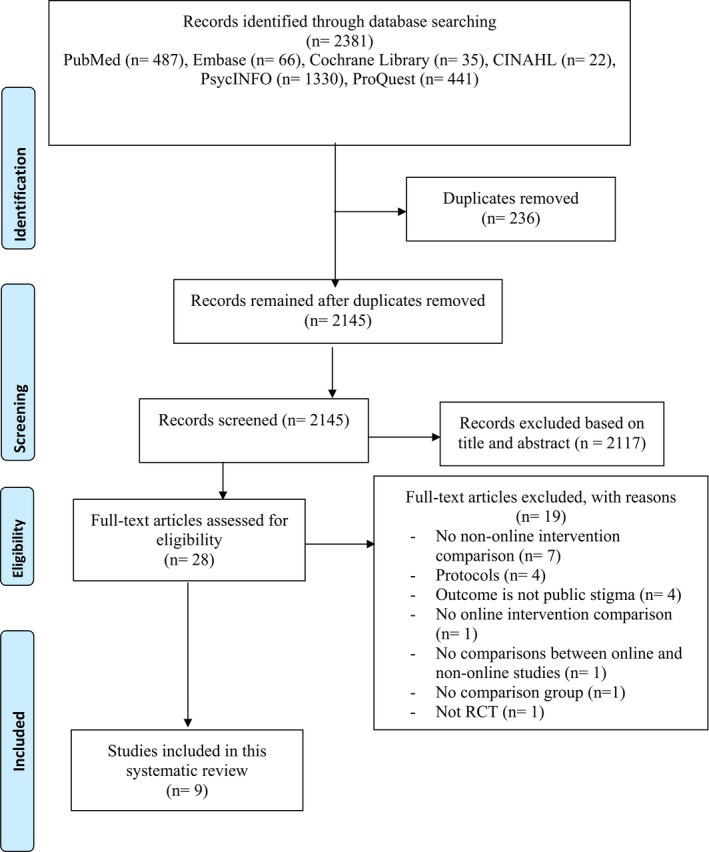
PRISMA flowchart.

Of the 2,381 studies retrieved from the databases, 173 duplicates were removed. Upon screening of the titles and abstracts, another 2117 studies were removed. For the remaining 28 studies, their full texts were retrieved, from which 19 were removed with reasons, leaving nine articles in the review (Figure 1 for PRISMA flowchart).

### Characteristics of the included studies

The selected studies were published from 2004 to 2019: five were conducted in Australia (Griffiths *et al*. 2016; Griffiths *et al*. 2004; Jorm *et al*. 2010; Kiropoulos *et al*. 2011; Shann *et al*. 2019), one in Russia (Finkelstein *et al*. 2007), one in the USA (Hamblen *et al*. 2019), one in the United Kingdom (UK) (Davies *et al*. 2018), and one in Canada (Moll *et al*. 2018). Their sample sizes ranged from 55 (Davies *et al*. 2018) to 525 (Griffiths *et al*. 2004), with a median of 196 (Table 1).

**Table 1:** Selected characteristics of included studies

Author	Year	Country	Aim of study	Study design	Sample size	Groups	Measure of stigma	Results
Shann *et al*.	2019	Australia	To investigate whether an online intervention could reduce leaders’ depression‐related stigma and develop their understanding and skills in relation to managing depression in the workplace	Longitudinal	196	Online: brief online workplace mental health intervention Non‐online: Wait‐List Control	Managerial Stigma Toward Employee Depression Scale (Affective, Cognitive, and Behavioural Stigma)	1‐week post‐test: A statistically significant difference in survey stigma was found between experimental and control groups, *V* = 0.09, *F*(3, 189) = 6.26, *P* < 0.001, η^2^ = .09 Affective stigma was significantly different between groups, *F*(1, 191) = 14.55, *P* < 0.001, η^2^ = 0.07; estimated marginal means indicated that the experimental group had lower affective stigma scores (*M* = 9.42, *SEM* = 0.24) at postsurvey compared with the control group (*M* = 10.51, *SEM* = 0.16) Behavioural stigma showed a significant group effect, *F*(1, 191) = 10.04, *P* = 0.002, η^2^ = 0.05. The experimental group had a lower behavioural stigma score (*M* = 6.22, *SEM* = .20) postsurvey than the control group (*M* = 6.99, *SEM* = .14) No statistical difference was found on the cognitive stigma means between the experimental group (*M* = 6.05, *SEM* = 0.18) and control group (*M* = 6.40, *SEM* = 0.13), *F*(1, 191) = 2.52, *P* = 0.11, η^2^ = 0.01 6‐month follow‐up: Affective stigma scores were significantly different between groups, *F*(1, 144) = 4.02, *P* = 0.04, η^2^ = 0.03. Estimated marginal means for the treatment group (*M* = 9.42, *SEM* = 0.32) were lower than that in the control group (*M* = 10.19, *SEM* = 0.22). No significant difference was found for the follow‐up behavioural stigma scores, *F*(1, 144) = 3.10, *P* = 0.08, η^2^ = 0.02
Finkelstein *et al*.	2007	Russia	To compare sustainability of the effect of two antistigma education programs	Longitudinal	91	Online: anti‐stigma web‐based program Non‐online: Brochure, Control (no‐intervention)	Bogardus Social Distance Scale (BSDS) for emotional and partly behavioural component of stigma and Community Attitudes toward the Mentally Ill (CAMI) for cognitive component of psychiatric stigma	Post‐test: In the program group, the level of stigma decreased from 18.8 ± 3.8 to 14.2 ± 4.6 points (BSDS, *t*‐test for paired samples *P* < 0.0001) and from 24.0 ± 5.0 to 15.8 ± 4.6 points (CAMI, *t*‐test for paired samples *P* < 0.0001) In the reading group, the level of stigma dropped from 18.5 ± 3.9 to 15.3 ± 4.4 points (BSDS, *t*‐test for paired samples *P* < 0.0001) and from 24.1 ± 6.1 to 20.3 ± 6.4 points (CAMI, *t*‐test for paired samples *P* < 0.0001) 6‐month follow‐up: There was no difference in CAMI scores between the computer, reading, and control groups (according to ANOVA, *P* = 0.31) There was a significant difference in BSDS scores, with the lowest level of stigma in the program group (ANOVA *P* = 0.03). In the program group, the level of stigma was significantly lower than at baseline (*t*‐test, two‐sample assuming equal variances CAMI *P* < 0.001, BSDS *P* = 0.02)
Kiropoulos *et al*.	2011	Australia	To investigate the effects of Multicultural Information on Depression Online (MIDonline), an Internet‐based multilingual depression‐specific information resource, on depression literacy, depression stigma, and depressive symptoms in Greek‐born and Italian‐born immigrants to Australia	Longitudinal	202	Online: MIDonline Non‐online: Depression interview control group	Depression Stigma Scale	Post‐test: MIDonline was associated with lower postintervention personal stigma scores than the control group, *F*(1,178) = 38.75, *P* < 0.001. 1‐week follow‐up: MIDonline was associated with lower follow‐up personal stigma scores than the control group, *F*(1,176) = 11.08, *P* = 0.001). A further ANCOVA of the follow‐up personal stigma measures controlling for postintervention personal stigma levels indicated that there was a trend toward a small reduction in the effect at follow‐up, *F*(1,176) = 3.65, *P* < 0.06
Jorm *et al*.	2010	Australia	To evaluate the effects of Mental Health First Aid training delivered by e‐learning on knowledge about mental disorders, stigmatizing attitudes and helping behaviour	Longitudinal	262	Online: e‐learning CD Non‐online: Printed manual, Waiting‐List Control	Subscales of The Depression Stigma Scale – Personal subscale (DSS‐Personal) and The Depression Stigma Scale – Perceived subscale (DSS‐Perceived)	1‐month post‐test: The e‐learning CD had positive effects compared to waiting list controls on personal stigma regarding both depression (OR = 8.65, 95%CI 2.30–32.5) and schizophrenia (OR = 24.31, 95%CI 5.91–99.92) The manual had positive effects compared to waiting list controls on personal stigma regarding both depression (OR = 13.68, 95%CI 3.54–52.85) and schizophrenia (OR = 5.28, 95%CI 1.50–18.52) The e‐learning CD had positive effects compared to the manual on personal stigma regarding schizophrenia (OR = 4.61, 95%CI 1.11–19.15) The e‐learning CD had greater reduction effects compared to the waiting list controls on social distance regarding schizophrenia (OR = 0.05, 95%CI 0.01–0.26) The e‐learning CD had greater reduction effects compared to the manual on social distance regarding schizophrenia (OR = 0.10, 95%CI 0.02–0.46) 6‐month follow‐up: The e‐learning CD had positive effects compared to waiting list controls on personal stigma regarding both depression (OR = 35.73, 95%CI 7.25–176.18) and schizophrenia (OR = 13.18, 95%CI 3.52–49.33) The manual had positive effects compared to waiting list controls on personal stigma regarding depression (OR = 12.04, 95%CI 3.24‐44.69) The e‐learning CD had positive effects compared to the manual on personal stigma regarding schizophrenia (OR = 4.40, 95%CI 1.17–16.54) The e‐learning CD had greater reduction effects compared to the waiting list controls on social distance regarding schizophrenia (OR = 0.09, 95%CI 0.02–0.42)
Hamblen *et al*.	2019	USA	To examine the impact of AboutFace on stigma, attitudes toward seeking mental health services, and PTSD mental health service use	Repeated Measures	60	Online: AboutFace Non‐online: Educational booklet about PTSD treatment (Usual Care)	The Endorsed and Anticipated Stigma Inventory (EASI)	All veterans reported non‐significantly improved attitudes toward mental illness, *F*(46) = 2.56, *P* = 0.12, from baseline (*M* = 59.5, *SD* = 27.9) to the 2‐week follow‐up (*M* = 56.0, *SD* = 31.7). There were no significant between‐group differences, *F*(45) = 2.14, *P* = 0.15, although the feasibility trial was not powered for this analysis
Griffiths *et al*.	2004	Australia	To investigate the effects on Stigma of two internet depression sites	Repeated Measures	525	Online: BluePages (web‐based depression literacy intervention), MoodGYM (web‐based cognitive–behavioural intervention) Non‐online: Control (weekly contact with an interviewer)	Personal and Perceived Depression Stigma Scale (18‐item)	An analysis of the change in stigma over time demonstrated a significant main effect for intervention, *F*(2,522) = 4.36, *P* = 0.013. In particular, stigma reduction was significantly greater in both the BluePages (mean difference 0.94, 95%CI 0.07–1.82, *P* = 0.031) and MoodGYM (mean difference 0.90, 95%CI 0.043–1.75, *P* = 0.036) groups than in the control condition after Bonferroni’s correction There was no significant difference between the two websites in stigma reduction (mean difference 0.04, 95%CI 70.83‐0.92, *P = *1.0)
Griffiths *et al*.	2016	Australia	To investigate the effectiveness of Mental Health Guru (MH‐Guru), a brief online universal workplace educational program about depression and generalised anxiety	Longitudinal	507	Online: MH‐Guru Non‐online: Waiting‐List Control	The Depression Stigma Scale – Personal subscale (DSS‐Personal) and The Generalised Anxiety Stigma scale – Personal subscale (GASS‐Personal)	1‐week post‐test: Significant interactions were found for both personal depression stigma (DSS‐personal) and generalised anxiety disorder stigma (GASS‐personal). The custom contrasts revealed a decline in depression stigma scores, *t*(421) = 6.4, *P* < 0.001, and anxiety stigma scores, *t*(416.6) = 5.5, *P* < 0.001, for MH‐Guru compared to control group participants from baseline to post‐test There were moderate between group effect sizes for depression (d = −0.56) and anxiety stigma at post‐test (d = −0.42), where the negative effect corresponded to a reduction in stigma for the MH‐Guru group 6‐month follow‐up: The custom contrasts revealed a decline in depression stigma scores, *t*(339.8) = 2.8, *P* = 0.005, and anxiety stigma scores, *t*(326.3) = 4.1, *P* < 0.001, for MH‐Guru compared to control group participants from baseline to 6‐month follow‐up There were moderate between group effect sizes for depression (*d* = −0.47) and anxiety (*d* = −0.42) stigma at 6 months, where the negative effect corresponded to a reduction in stigma for the MH‐Guru group
Davies *et al*.	2018	UK	To evaluate the MHFA eLearning course in UK medical students	Repeated Measures	55	Online: MHFA eLearning course Non‐online: Control (no‐intervention)	The personal stigma subscale of the Depression Stigma Scale (DSS)	Stigma reduced over time, *F*(1,35) = 8.38, *P* = 0.007, and there was an interaction between time and group, *F*(1,35) = 4.41, *P* = 0.043) Stigma significantly declined in the intervention group, *Z* = −2.30, *P* = 0.021, but not in controls, *Z* = −0.748, *P* = 0.45
Moll *et al*.	2018	Canada	To evaluate whether a contact‐based workplace education program was more effective than standard mental health literacy training in promoting early intervention and support for healthcare employees with mental health issues	Longitudinal	192	Online Beyond Silence Non‐online: Mental First Health Aid program	Opening Minds Scale for Health Care providers	3‐month post‐test: In the stigma analysis, no interactions for treatment arm by time were observed (beta = −0.21, *z* = −0.22, *P* = 0.83) 6‐month follow‐up: A possible trend for superior outcomes for Beyond Silence was seen at 6 months (beta = 1.72, *z* = 1.70, *P* = 0.089). To explore whether the anti‐stigma effects of Beyond Silence might be more persistent than those of MHFA, a model describing changes from 3 to 6 months was fit, revealing a significant treatment by time interaction (beta = 1.89, *z* = 2.09, *P* = 0.037)

### Risk of bias within the studies

**Figure 2:**
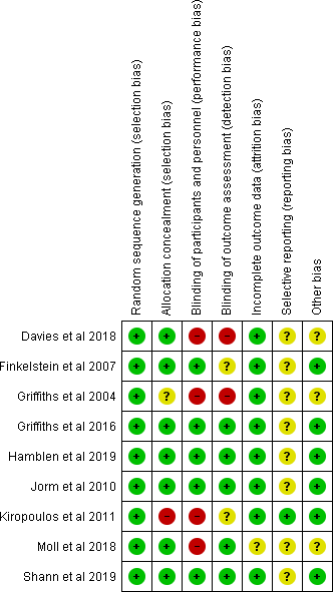
ROB summary.

Of the nine included studies (Figure 2), the majority were assessed to be of a low overall risk of bias; in almost each of them, the unclear risk was found to originate chiefly from one category (selective reporting). High risk of bias was found mainly in allocation concealment, blinding of participants and personnel, and outcome assessment.

### Primary outcome

#### Public stigma towards PMHCs

**Figure 3:**
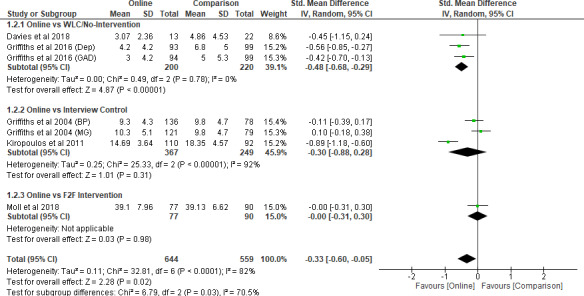
Intervention modality comparison (Stigma) – before removal.

**Figure 4:**
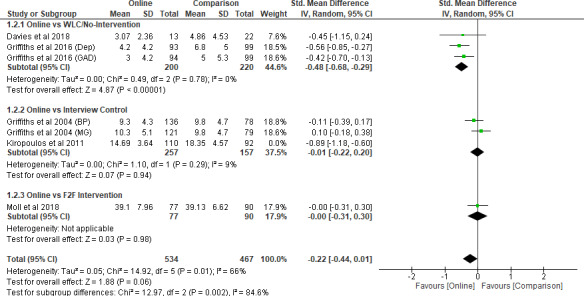
Intervention modality comparison (Stigma) –after removal.

A meta‐analysis was conducted on five studies (Davies *et al*. 2018; Griffiths *et al*. 2016; Griffiths *et al*. 2004; Kiropoulos *et al*. 2011; Moll *et al*. 2018) which included 1203 participants in the meta‐analysis. Two of the five studies each compared a group with an online intervention with a no‐intervention group or a wait‐list control (WLC) group (Davies *et al*. 2018; Griffiths *et al*. 2016). Two other studies each compared a group with an online intervention with a control group subjected to an interview (Griffiths *et al*. 2004; Kiropoulos *et al*. 2011). The last study compared a group with an online intervention with another with a face‐to‐face intervention (Moll *et al*. 2018). The combined standardized mean difference (SMD) from the five studies was –0.33 (95% CI –0.60 to –0.05, *P* = 0.02). The heterogeneity was *I* ^2^ = 82% (*P* < 0.001), alongside significant subgroup differences between three subgroups (*P* = 0.03) (Figure 3). As Kiropoulos *et al*. (2011) exhibited a much larger SMD of –0.89 (95% CI –1.18 to –0.60) than the two intervention groups from Griffiths *et al*. (2004), sensitivity analysis was conducted by excluding Kiropoulos *et al*. (2011). After removal, the combined SMD from the four studies stood at –0.22 (95% CI –0.44 to 0.01, *P* = 0.06). The heterogeneity thus declined to *I* ^2^ = 66% (*P* < 0.001), alongside significant subgroup differences between the three subgroups (*P* = 0.002) (Figure 4). This indicates that the group with the online interventions demonstrated a significant reduction of public stigma only when compared to the WLC or no‐intervention groups but not when compared to other non‐online intervention groups.

Four studies (Finkelstein *et al*. 2007; Hamblen *et al*. 2019; Jorm *et al*. 2010; Shann *et al*. 2019) could not be included in the meta‐analysis and were analysed narratively due to missing summary data (Table 1). A significant stigma‐reduction effects were found for affective [*F*(1, 191) = 14.55, *P* < 0.001, η^2^ = 0.07] and behavioural [*F*(1, 191) = 10.04, *P* = 0.002, η^2^ = 0.05] public stigma subscales for online interventions (Shann *et al*. 2019). Finkelstein *et al*. (2007) found significant stigma‐reduction effects for a group with an online intervention and for a reading group, both in comparison to a no‐intervention group (BSDS online and reading; *P* < 0.0001, CAMI online and reading; *P* < 0.0001) although no comparison was conducted between treatment groups. Likewise, in investigating stigmatization towards people with schizophrenia, Jorm *et al*. (2010) found significant stigma‐reduction effects for a group with an online intervention and for a reading group, both in comparison to the WLC group. Their further analysis found more substantial stigma‐reduction effects for the group with the online intervention than for the reading group (OR = 4.61, 95%CI 1.11–19.15). Finally, Hamblen *et al*. (2019) found no stigma‐reduction effects for both the group with an online intervention and the reading group, which might have been due to pre‐existing positive attitudes towards mental health treatment at the baseline among all participants. Thus, these findings collectively suggest that online interventions were at least as effective as face‐to‐face interventions in reducing public stigma towards PMHCs.

**Figure 5:**
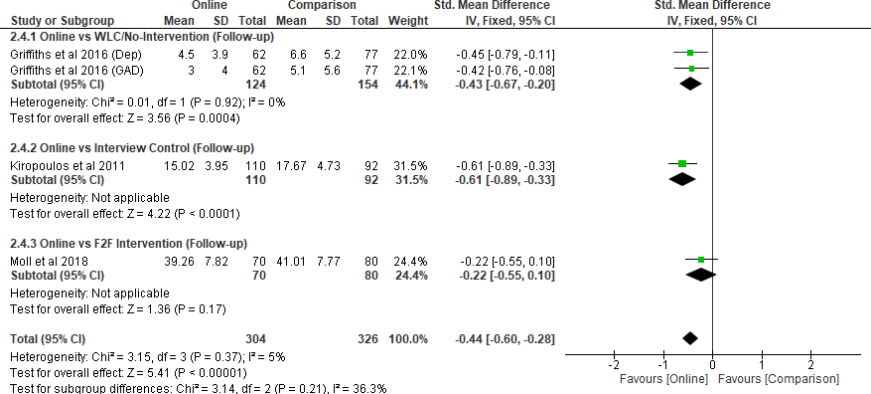
Intervention modality comparison (Stigma) – Long‐Term Effects.

A meta‐analysis was conducted on three studies (Griffiths *et al*. 2016; Kiropoulos *et al*. 2011; Moll *et al*. 2018) which included follow‐up assessments and long‐term effects of the interventions for stigma towards PMHCs. A total of 428 participants were included in the meta‐analysis. The combined SMD from the three studies stood at –0.44 (95% CI –0.60 to –0.28, *P* < 0.001). The heterogeneity was *I* ^2^ = 5% (*P* = 0.37) (Figure 5). This small‐to‐medium effect size suggests longitudinal stigma‐reduction effects of the online interventions. However, as the caveat is that only three out of five studies examined longer‐term stigma‐reduction effects in the meta‐analysis, this finding should be interpreted with caution.

Three studies (Finkelstein *et al*. 2007; Jorm *et al*. 2010; Shann *et al*. 2019) that included follow‐up assessments and long‐term effects of the interventions for stigma towards PMHCs were not included in the meta‐analysis due to missing summary data (Table 1). All three studies found significant stigma‐reduction effects in their online interventions as compared to reading, WLC, and no‐intervention groups. These findings collectively suggest potential longitudinal stigma‐reduction effects for such online interventions that may be worth further elucidation.

### Secondary outcomes

#### Stigma experienced by people with different mental health conditions

**Figure 6:**
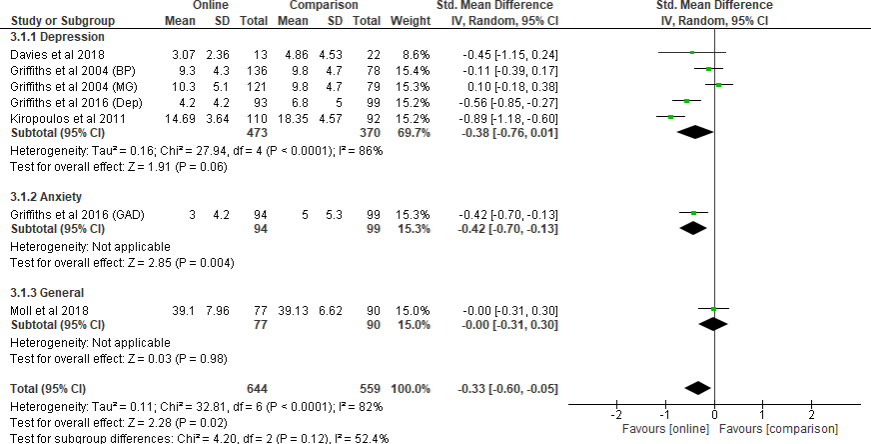
Mental health conditions comparison (Stigma) – before removal.

A meta‐analysis was conducted on five studies (Davies *et al*. 2018; Griffiths *et al*. 2016; Griffiths *et al*. 2004; Kiropoulos *et al*. 2011; Moll *et al*. 2018) according to the type of mental health conditions examined. Three studies examined the stigma towards people with depression (Davies *et al*. 2018; Griffiths *et al*. 2004; Kiropoulos *et al*. 2011). One study examined that towards people with any mental health conditions (Moll *et al*. 2018) and another study examined that towards people with either depression or anxiety (Griffiths *et al*. 2016). The pooled result for depression was –0.38 (95% CI –0.76 to 0.01, *P* = 0.06), with the heterogeneity being *I* ^2^ = 86% (*P* < 0.001). The result for anxiety consisting of only scores from Griffiths *et al*. (2016)was –0.42 (95% CI –0.70 to –0.13, *P* = 0.004). The result of general mental health conditions consisting of only scores from a sole study (Moll *et al*. 2018) was –0.00 (95% CI –0.31 to 0.30, *P* = 0.98). No significant subgroup differences were detected between the three types of mental health conditions (*P* = 0.12). Similarly, sensitivity analysis was conducted by excluding results from Kiropoulos *et al*. (2011). The differences regarding stigma reduction between the types of mental health conditions remained non‐significant after removal (*P* = 0.15) (Figure 6).

### Knowledge of mental health conditions

**Figure 7:**
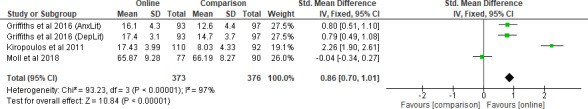
Knowledge of mental health conditions.

Only three studies (Griffiths *et al*. 2016; Kiropoulos *et al*. 2011; Moll *et al*. 2018) measured knowledge of mental health conditions. One study measured only the literacy on depression (Kiropoulos *et al*. 2011), another measured the literacy on both depression and anxiety (Griffiths *et al*. 2016), and the third measured the knowledge of general mental health (Moll *et al*. 2018). These differences could account for the substantial heterogeneity (*I* ^2^ = 97%, *P* < 0.001) which could otherwise not be pooled. Griffiths *et al*. (2016) reported a MD of 0.80 (95% CI 0.51 to 1.10, *P* < 0.001) for the literacy on anxiety and a MD of 0.79 (95% CI 0.49 to 1.08, *P* < 0.001) for the literacy on depression between the group with an online intervention and the WLC group. Kiropoulos *et al*. (2011) reported a MD of 2.26 (95% CI 1.90 to 2.61, *P* < 0.001) between the group with an online intervention and interview control group. Moll *et al*. (2018) reported a MD of –0.04 (95% CI −0.34 to 0.27, *P* = 0.81) between the group with an online intervention and that with a face‐to‐face intervention (Figure 7).

### Desire for social contact with PMHCs

Only one study (Jorm *et al*. 2010) measured the desire for social distancing (contact) with PMHCs. The online intervention was found to produce greater reductions in the desire for social distancing (improvements in desire for social contact) towards PMHCs as compared to both the reading (OR = 0.10, 95%CI 0.02–0.46) and WLC (OR = 0.05, 95% CI 0.01–0.26) groups. However, this effect was not maintained at follow‐ups (OR = 0.09, 95% CI 0.02–0.42) (Table 1).

## Discussion

This review aimed to examine the effects of online and face‐to‐face anti‐stigma interventions in reducing public stigma towards people with various mental health conditions. Nine studies comparing an online stigma‐reduction intervention with a non‐online one or a control group were included in the systematic review. However, only five studies were included in the meta‐analysis as the rest of the studies had missing or incomplete data. The said five studies included an aggregate of 1203 participants (644 online, 559 comparison) while the four excluded ones had an aggregate of 713 participants (305 online, 410 comparison). Overall, results from pooled values of post‐programme data and studies excluded from the meta‐analysis revealed that participants in the online intervention groups demonstrated a significant reduction of public stigma in comparison to non‐online, WLC, and no‐intervention groups. The small effect sizes observed are consistent with those for other public stigma‐reduction interventions (Corrigan *et al*. 2012) and subgroup differences concerning the modality of interventions remained significant for the pooled values. Findings from our study are supported by other evidence‐based online anti‐stigma interventions, which have proved a promising avenue for removing barriers to accessing help for mental health symptoms among PMHCs (Nickerson *et al*. 2020). Furthermore, according to the social‐cognitive model (Corrigan *et al*. 2001), the reduction of stigma could be a result of the modification of erroneous social beliefs such as stereotypes and attitudes towards PMHCs (Pedersen *et al*. 2011). Therefore, such findings suggest that online interventions were as effective as non‐online ones in reducing public stigma towards PMHCs.

One noteworthy finding concerns the absence of between‐group differences in the effectiveness of the interventions for the various mental health conditions studied. This suggests that online interventions targeting stigma towards any form of such conditions will likely exhibit similar effectiveness (Hanisch *et al*. 2016). As aforesaid, no meta‐analysis could be performed for the effects of the interventions on the knowledge of mental health conditions and the desire for social contact with PMHCs, given the heterogeneity of the studies and their missing data. However, individual results from the few studies examining these outcomes indicate possible benefits of online interventions and warrant further research. This review contributes to the findings of previous systematic reviews on the effectiveness of general stigma interventions (Corrigan *et al*. 2012; Griffiths *et al*. 2014; Yamaguchi *et al*. 2013b) in its examination of interventional modality in public stigma research. Traditional interventions typically use educational (delivering content on mental health conditions and challenging myths) and social contact‐based approaches (involving personal contact or presentations by PMHCs) that have been found effective in reducing public stigma (Corrigan *et al*. 2012; Griffiths *et al*. 2014; Yamaguchi *et al*. 2013b). Based on the intergroup contact theory (Allport 1955), the premise for social contact‐based approaches involves invoking greater familiarity and a more positive appraisal of PMHCs (Holmes *et al*. 1999), therefore raising the possibility that online interventions may diminish the effectiveness that these in‐person interventions offer.

However, social contact‐based interventions through video recordings have been found equally effective in achieving de‐stigmatization effects (Brown 2020; Janoušková *et al*. 2017). As all nine studies in this review have integrated either one or both approaches in their online interventions, it is unsurprising that a significant stigma‐reduction result was found when compared to WLC or no‐intervention groups. This may indicate that traditional stigma interventions can be successfully transferred to online platforms while retaining their clinical utility (Sampogna *et al*. 2017). Nonetheless, online interventions come with challenges: high drop‐out rates have been observed, especially for interventions spanning substantial periods of time (Donkin & Glozier 2012). Pedersen *et al*. (2019) have suggested that the highest risk of drop‐outs from any programme would happen in the beginning when attrition resulted from a reduction of online activities from participants over time. Hence, programme coordinators should discern abnormal decreases in online activities as an indicator reflective of potential attrition and pre‐empt it by initiating re‐engagement. Furthermore, helping participants to identify and incorporate intrinsic motivations such as noticing their own improvement has been found to foster a sense of duty to self and personal empowerment: this component in the programme can further encourage adherence (Donkin & Glozier 2012).

The dearth of research comparing online and non‐online public stigma interventions in conjunction with the missing summary data for multiple secondary outcomes prevented further conclusive remarks regarding the effectiveness of online and face‐to‐face interventions. However, our findings suggest that the online interventions afforded public stigma‐reduction effects (Roslee & Goh 2020) that may last well beyond their initial completion. This bears critical implications for the future delivery of such interventions: the rapid proliferation of internet users (International Telecommunication Union 2020) and the evolving COVID‐19 pandemic may increase the acceptability of online interventions (Wind *et al*. 2020). It may follow that such interventions may be delivered to a larger population at a lower cost (Rogers 2018) without compromising their quality and effectiveness.

However, mental health practitioners need to be vigilant in noting any potential unintended effects from the interventions that could eventually reinforce the public stigma which they are designed to address. Although the use of online anti‐stigma interventions has overcome geographical, mobility, and time constraints, potential costs on the users and their social networks have to be considered. PMHCs already facing public stigma may feel further stigmatized when excluded from the mainstream healthcare delivery system (Corrigan & Rao 2012; Rüsch *et al*. 2005). It is, therefore, important for researchers to include theoretical underpinnings that can provide an empirical evaluation on their chosen online anti‐stigma interventions in place of non‐online ones (Griffiths *et al*. 2006). Finally, having randomized control trials with non‐online anti‐stigma interventions will improve the evaluation of Internet‐based interventions as an effective prospective intervention.

### Strength and limitations

Through our timely evaluation of the effectiveness of online anti‐stigma interventions, healthcare providers, not‐for‐profit organizations, and mental health advocacy groups looking to implement such interventions have been provided with an overview of the advantages and disadvantages. This allows them to make an informed decision when choosing the most appropriate modality for their specific needs. However, some limitations are of note, such as the small number of studies comparing online and non‐online interventions addressing public stigma; substantial heterogeneity in some analyses due to the variations of the contents of the interventions, the comparator (or usual care) and the instruments used in measuring the outcome; and missing summary data in many studies. As a result, only a small number of trials were included in the meta‐analysis (four trials), thus limiting the ability for further conclusions to be drawn. Despite that, this review still provides a valuable foundation for future research examining the impact of the modality on interventions addressing public stigma. In this review, the pre‐defined protocol (Goh *et al*. 2020) was adhered to with some minor alterations to the secondary outcomes. The longitudinal effect of the stigma interventions was considered as an extension of the primary outcome while the pre‐defined subgroup comparing population groups was modified to examine different mental health conditions.

## Conclusions

The current review of nine studies provides evidence that online interventions are comparatively effective in reducing public stigma towards PMHCs as non‐online interventions. Future replications and research on this topic are required to establish conclusive evidence for their effectiveness. Additionally, future research may consider examining the effectiveness of online interventions on not only stigma‐related areas (such as knowledge and desire for social contact) but also the type of stigmatized mental health conditions; these aspects have remained unclear due to the limited studies on this area of research.

## Relevance to Clinical Practice

This review presents some evidence that the effectiveness of online interventions in reducing public stigma towards PMHCs would persist even after the conclusion of the intervention. The effectiveness of online interventions did not differ based on the types of mental health conditions. As the interventions reviewed focused on stigma towards PMHCs, organizations, and groups with an interest in mental health may consider adopting online interventions suitable for their target audience. Due to the small effect sizes found in the studies in this review and other interventions addressing public stigma (Corrigan *et al*. 2012), further developments of stigma interventions, regardless of the modality, may also be warranted for greater efficacy in this field.

## Ethical Approval

Not applicable.

## Data Availability

Data are openly available in a public repository that issues datasets with DOIs.
